# Supplementary material for: Motor unit mechanisms of speed control in mouse locomotion
Source: eLife. 2026 Oct 22;14:RP105829. doi: 10.7554/eLife.105829 (PMC13690341; doi:10.7554/eLife.105829)
Supplement: Supplementary file 1 [file elife-105829-mdarchecklist1.pdf]

eLife

Materials Design Analysis Reporting (MDAR) Checklist for Authors

The MDAR framework establishes a minimum set of requirements in transparent reporting mainly applicable to studies in the life sciences.

eLife asks authors to **provide detailed information within their article** to facilitate the interpretation and replication of their work. Authors can also upload supporting materials to comply with relevant reporting guidelines for health-related research (see EQUATOR Network), life science research (see the BioSharing Information Resource), or animal research (see the ARRIVE Guidelines and the STRANGE Framework; for details, see *eLife*'s Journal Policies). Where applicable, authors should refer to any relevant reporting standards materials in this form.

For all that apply, please note **where in the article** the information is provided. Please note that we also collect information about data availability and ethics in the submission form.

Materials:

Newly created materials	Indicate where provided: section/figure legend	N/A
The manuscript includes a dedicated "materials availability statement" providing transparent disclosure about availability of newly created materials including details on how materials can be accessed and describing any restrictions on access.		X

Antibodies	Indicate where provided: section/figure legend	N/A
For commercial reagents, provide supplier name, catalogue number and RRID, if available.		X

DNA and RNA sequences	Indicate where provided: section/figure legend	N/A
Short novel DNA or RNA including primers, probes: Sequences should be included or deposited in a public repository.		X

Cell materials	Indicate where provided: section/figure legend	N/A
Cell lines: Provide species information, strain. Provide accession number in repository OR supplier name, catalog number, clone number, OR RRID.		X
Primary cultures: Provide species, strain, sex of origin, genetic modification status.		X

Experimental animals	Indicate where provided: section/figure legend	N/A
Laboratory animals or Model organisms: Provide species, strain, sex, age, genetic modification status. Provide accession number in repository OR supplier name, catalog number, clone number, OR RRID.	Methods section, Table 1	
Animal observed in or captured from the field: Provide species, sex, and age where possible.		X

Plants and microbes	Indicate where provided: section/figure legend	N/A
Plants: provide species and strain, ecotype and cultivar where relevant, unique accession number if available, and source (including location for collected wild specimens).		X
Microbes: provide species and strain, unique accession number if available, and source.		X

Human research participants	Indicate where provided: section/figure legend) or state if these demographics were not collected	N/A
If collected and within the bounds of privacy constraints report on age, sex, gender and ethnicity for all study participants.		**X**

Design:

Study protocol	Indicate where provided: section/figure legend	N/A
If the study protocol has been pre-registered, provide DOI. For clinical trials, provide the trial registration number OR cite DOI.		X

Laboratory protocol	Indicate where provided: section/figure legend	N/A
Provide DOI OR other citation details if detailed step-by-step protocols are available.		X

**Experimental study design (statistics details) ***		
**For in vivo studies: State whether and how the following have been done**	**Indicate where provided: section/figure legend. If it could have been done, but was not, write "not done"**	**N/A**
Sample size determination		
Randomization	**Methods: Behavioral methods and data collection.** Locomotor-speed trials were presented in a pseudo-random order within blocks for five of six mice; this was done to sample the full speed range while reducing fatigue/order effects. There was no treatment-group allocation.	X
Blinding	N/A (no treatment vs non-treatment groups)	X
Inclusion/exclusion criteria	**Methods: Behavioral methods and data collection;** Electromyography. Kinematic points with DeepLabCut confidence below 90% were excluded/interpolated; strides were required to include footstrike and liftoff during forward movement; strides with stride duration, stance duration, swing duration, body velocity, or acceleration outside the respective 95% confidence interval were excluded, leaving ~80% of strides per animal. Motor-unit isolation required ≤2% inter-spike intervals violating a 1 ms refractory limit, with manual review of cross-correlograms.	X

**Sample definition and in-laboratory replication**	**Indicate where provided: section/figure legend**	**N/A**
State number of times the experiment was replicated in the laboratory.	**Methods: Behavioral methods and data collection; Table 1.** The experiment was replicated across 6 mice. Five mice had 23–31 one-minute treadmill trials, and the sixth had 14 trials; five of six mice contributed >1000 analyzed strides, with five having 2600–3600 analyzed strides.	

Define whether data describe technical or biological replicates.	**Methods: Behavioral methods and data collection; Table 1.** Biological replicates are independent mice and, for motor-unit-level analyses, independently isolated motor units. Multiple trials and strides within the same mouse/motor unit are repeated within-subject observations rather than independent biological replicates.	

Ethics	Indicate where provided: section/submission form	N/A
Studies involving human participants: State details of authority granting ethics approval (IRB or equivalent committee(s), provide reference number for approval.		X
Studies involving experimental animals: State details of authority granting ethics approval (IRB or equivalent committee(s), provide reference number for approval.	Emory University IACUC protocol approval #201700359.	
Studies involving specimen and field samples: State if relevant permits obtained, provide details of authority approving study; if none were required, explain why.		X

Dual Use Research of Concern (DURC)	Indicate where provided: section/submission form	N/A
If study is subject to dual use research of concern regulations, state the authority granting approval and reference number for the regulatory approval.		X

Analysis:

Attrition	Indicate where provided: section/figure legend	N/A
Describe whether exclusion criteria were pre-established. Report if sample or data points were omitted from analysis. If yes, report if this was due to attrition or intentional exclusion and provide justification.		X

Statistics	Indicate where provided: section/figure legend	N/A

Describe statistical tests used and justify choice of tests.	**Results; Methods: Data analysis.** Statistical analyses included Hartigan's dip test for multimodality of recruitment-probability distributions; two-sample Kolmogorov–Smirnov tests for comparing distributions of timing/ISI-related measures between muscles; Wilcoxon signed-rank tests for paired motor-unit comparisons; permutation tests for speed-dependent changes and cross-muscle differences in type 2 regression slopes; and a likelihood-based joint model of recruitment probability and firing rate. Non-parametric and permutation-based tests were appropriate because many comparisons were across motor units with modest sample sizes and without assuming normality.	

**Data availability**	**Indicate where provided: section/submission form**	**N/A**
For newly created and reused datasets, the manuscript includes a data availability statement that provides details for access (or notes restrictions on access).	Data availability statement	
When newly created datasets are publicly available, provide accession number in repository OR DOI and licensing details where available.	"A data archive including processed data and raw EMG/kinematic data for each of six locomoting mice has been included is available at DOI: 10.5061/dryad.wdbrv162w"	
If reused data is publicly available provide accession number in repository OR DOI, OR URL, OR citation.		X

**Code availability**	**Indicate where provided: section/figure legend**	**N/A**
For any computer code/software/mathematical algorithms essential for replicating the main findings of the study, whether newly generated or re-used, the manuscript includes a data availability statement that provides details for access or notes restrictions.	"A data archive including processed data and raw EMG/kinematic data for each of six locomoting mice has been included is available at DOI: 10.5061/dryad.wdbrv162w"	

Where newly generated code is publicly available, provide accession number in repository, OR DOI OR URL and licensing details where available. State any restrictions on code availability or accessibility.	All code for plotting figures is provided at DOI: 10.5061/dryad.wdbrv162w	
If reused code is publicly available provide accession number in repository OR DOI OR URL, OR citation.	**Methods: Behavioral methods and data collection; Electromyography.** Reused software includes DeepLabCut for tracking, Bonsai/Arduino components for acquisition synchronization, and Kilosort 2.5 for spike sorting; these are cited in the manuscript.	

Reporting:

The MDAR framework recommends adoption of discipline-specific guidelines, established and endorsed through community initiatives.

**Adherence to community standards**	**Indicate where provided: section/figure legend**	**N/A**
State if relevant guidelines (e.g., ICMJE, MIBBI, ARRIVE, STRANGE) have been followed, and whether a checklist (e.g., CONSORT, PRISMA, ARRIVE) is provided with the manuscript.		X
