# Supplementary material for: Motor unit mechanisms of speed control in mouse locomotion
Source: eLife. 2026 Oct 22;14:RP105829. doi: 10.7554/eLife.105829 (PMC13690341; doi:10.7554/eLife.105829)
Supplement: Peer review history

# Editor report: eLife Assessment

**DOI:** 10.7554/eLife.105829.4.sa0

**Authors:** Juan Alvaro Gallego

**Affiliations:** https://ror.org/03g001n57 Champalimaud Foundation Portugal

**Keywords:** Convincing, Valuable

This **valuable** study characterises the activity of motor units from two of the three anatomical subdivisions ("heads") of the triceps muscle while mice walked on a treadmill at various speeds. Altogether, this is the most thorough characterisation of motor unit activity in walking mice to date, providing **convincing** evidence for probabilistic recruitment of motor units that differed between the two heads.

# Referee report: Reviewer #1 (Public review)

**DOI:** 10.7554/eLife.105829.4.sa1

[Editors' note: this version has been assessed by the Reviewing Editor without further input from the original reviewers. The authors have addressed the comments raised in the previous round of review.]

Summary:

Here, the authors have addressed the recruitment and firing patterns of motor units (MUs) from the long and lateral heads of triceps in the mouse. They used their newly developed Myomatrix arrays to record from these muscles during treadmill locomotion at different speeds, and they used template-based spike sorting (Kilosort) to extract units. Between MUs from the two heads, the authors observe differences in their firing rates, recruitment probability, phase of activation within the locomotor cycle and interspike interval patterning. Examining different walking speeds, the authors find increases in both recruitment probability and firing rates as speed increases. The authors also observed differences in the relation between recruitment and the angle of elbow extension between motor units from each head. These differences indicate meaningful variation between motor units within and across motor pools, and may reflect the somewhat distinct joint actions of the two heads of triceps.

Strengths:

The extraction of MU spike timing for many individual units is an exciting new method that has great promise for exposing the fine detail in muscle activation and its control by the motor system. In particular, the methods developed by the authors for this purpose seem to be the only way to reliably resolve single MUs in the mouse, as the methods used previously in humans and in monkeys (e.g. Marshall et al. Nature Neuroscience, 2022) do not seem readily adaptable for use in rodents.

The paper provides a number of interesting observations. There are signs of interesting differences in MU activation profiles for individual muscles here, consistent with those shown by Marshall et al. It is also nice to see fine scale differences in the activation of different muscle heads, which could relate to their partially distinct functions. The mouse offers greater opportunities for understanding the control of these distinct functions, compared to the other organisms in which functional differences between heads have previously been described.

The Discussion is very thorough, providing a very nice recounting of a great deal of relevant previous results.

# Referee report: Reviewer #2 (Public review)

**DOI:** 10.7554/eLife.105829.4.sa2

The present study, led by Thomas and collaborators, aims to characterise the firing activity of individual motor units in mice during locomotion. To achieve this, the team implanted small arrays of eight electrodes into two heads of the triceps and performed spike sorting using a custom implementation of Kilosort. Concurrently, they tracked the positions of the shoulder, elbow, and wrist using a single camera and a markerless motion capture algorithm (DeepLabCut). Repeated one-minute recordings were conducted in six mice across five speeds, ranging from 10 to 27.5 cm-1.

From these data, the authors demonstrate that:

- Their recording method and adapted spike-sorting algorithm enable robust decoding of motor unit activity during rapid movements.

- Identified motor units tend to be recruited during a subset of strides, with recruitment probability increasing with speed.

- Motor units within individual heads of the triceps likely receive common synaptic inputs that correlate their activity, whereas motor units from different heads exhibit distinct behaviour.

The authors conclude that these differences arise from the distinct functional roles of the muscles and the task constraints (i.e., speed).

Strengths:

- The novel combination of electrode arrays for recording intramuscular electromyographic signals from a larger muscle volume, paired with an advanced spike-sorting pipeline capable of identifying motor unit populations.

- The robustness of motor unit decoding during fast movements.

Weaknesses:

- The data do not clearly indicate which motor units were sampled from each pool, leaving uncertainty as to whether the sample is biased towards high-threshold motor units or representative of the entire pool.

- The results largely confirm the classic physiological framework of motor unit recruitment and rate coding, offering limited new insights into motor unit physiology.

Comments on previous version:

I would like to thank the authors for their thorough and insightful revisions. I am particularly pleased with the inclusion of the new analyses demonstrating the robustness of motor unit decoding, as well as the improved transparency regarding spike-sorting yield for each muscle and animal. Additionally, the new analyses illustrating that recruitment within muscle heads is consistent with the presence of common synaptic inputs and orderly recruitment significantly strengthen the manuscript.

# Referee report: Reviewer #3 (Public review)

**DOI:** 10.7554/eLife.105829.4.sa3

Summary:

Using the approach of Myomatrix recording, the authors report that (1) motor units are recruited differently in the two types of muscles and (2) individual units are probabilistically recruited during the locomotion strides, whereas the population bulk EMG has a more reliable representation of the muscle. Third, the recruitment of units was proportional to walking speed.

Strengths:

The new technique provides a unique dataset, and the data analysis is convincing and well-executed.

Weaknesses:

After the revision, I no longer see any apparent weaknesses in the study.

# Author comment: Author response

**DOI:** 10.7554/eLife.105829.4.sa4

**Authors:** Kyle Thomas, Rhuna Gibbs, Hugo G Marques, Megan R Carey, Samuel J Sober

**Affiliations:** https://ror.org/02j15s898 The Wallace H. Coulter Department of Biomedical Engineering Atlanta United States; https://ror.org/03czfpz43 Emory University Atlanta United States; https://ror.org/03g001n57 Champalimaud Foundation Lisbon Portugal; https://ror.org/052gg0110 University of Oxford Oxford United Kingdom; https://ror.org/03czfpz43 Emory University Atlanta United States

The following is the authors’ response to the previous reviews

**Public Reviews:**

**Reviewer #1 (Public review):**

Summary:

Here, the authors have addressed the recruitment and firing patterns of motor units (MUs) from the long and lateral heads of the triceps in the mouse. They used their newly developed Myomatrix arrays to record from these muscles during treadmill locomotion at different speeds, and they used template-based spike sorting (Kilosort) to extract units. Between MUs from the two heads, the authors observed differences in their firing rates, recruitment probability, phase of activation within the locomotor cycle, and interspike interval patterning. Examining different walking speeds, the authors find increases in both recruitment probability and firing rates as speed increases. The authors also observed differences in the relation between recruitment and the angle of elbow extension between motor units from each head. These differences indicate meaningful variation between motor units within and across motor pools and may reflect the somewhat distinct joint actions of the two heads of triceps.

Strengths:

The extraction of MU spike timing for many individual units is an exciting new method that has great promise for exposing the fine detail in muscle activation and its control by the motor system. In particular, the methods developed by the authors for this purpose seem to be the only way to reliably resolve single MUs in the mouse, as the methods used previously in humans and in monkeys (e.g. Marshall et al. Nature Neuroscience, 2022) do not seem readily adaptable for use in rodents.

The paper provides a number of interesting observations. There are signs of interesting differences in MU activation profiles for individual muscles here, consistent with those shown by Marshall et al. It is also nice to see fine-scale differences in the activation of different muscle heads, which could relate to their partially distinct functions. The mouse offers greater opportunities for understanding the control of these distinct functions, compared to the other organisms in which functional differences between heads have previously been described.

The Discussion is very thorough, providing a very nice recounting of a great deal of relevant previous results.

We thank the Reviewer for these comments.

Weaknesses:

The findings are limited to one pair of muscle heads. While an important initial finding, the lack of confirmation from analysis of other muscles acting at other joints leaves the general relevance of these findings unclear.

The Reviewer raises a fair point. While outside the scope of this paper, future studies should certainly address a wider range of muscles to better characterize motor unit firing patterns across different sets of effectors with varying anatomical locations. Still, the importance of results from the triceps long and lateral heads should not be understated as this paper, to our knowledge, is the first to capture the difference in firing patterns of motor units across any set of muscles in the locomoting mouse.

While differences between muscle heads with somewhat distinct functions are interesting and relevant to joint control, differences between MUs for individual muscles, like those in Marshall et al., are more striking because they cannot be attributed potentially to differences in each head's function. The present manuscript does show some signs of differences for MUs within individual heads: in Figure 2C, we see what looks like two clusters of motor units within the long head in terms of their recruitment probability. However, a statistical basis for the existence of two distinct subpopulations is not provided, and no subsequent analysis is done to explore the potential for differences among MUs for individual heads.

We agree with the Reviewer and have revised the manuscript to better examine potential subpopulations of units within each muscle as presented in Figure 2C. We performed Hartigan’s dip test on motor units within each muscle to test for multimodal distributions. For both muscles, p > 0.05, so we can not reject the null hypothesis that the units in each muscle come from a multimodal distribution. However, Hartigan’s test and similar statistical methods have poor statistical power for the small sample sizes (n=17 and 16 for long and lateral heads, respectively) considered here, so the failure to achieve statistical significance might reflect either the absence of a true difference or a lack of statistical resolution.

Still, the limited sample size warrants further data collection and analysis since the varying properties across motor units may lead to different activation patterns. Given these results, we have edited the text as follows:

“A subset of units, primarily in the long head, were recruited in under 50% of the total strides and with lower spike counts (Figure 2C). This distribution of recruitment probabilities might reflect a functionally different subpopulation of units. However, the distribution of recruitment probabilities were not found to be significantly multimodal (p>0.05 in both cases, Hartigan’s dip test; Hartigan, 1985). However, Hartigan’s test and similar statistical methods have poor statistical power for the small sample sizes (n=17 and 16 for long and lateral heads, respectively) considered here, so the failure to achieve statistical significance might reflect either the absence of a true difference or a lack of statistical resolution.”

The statistical foundation for some claims is lacking. In addition, the description of key statistical analysis in the Methods is too brief and very hard to understand. This leaves several claims hard to validate.

We thank the Reviewer for these comments and have clarified the text related to key statistical analyses throughout the manuscript, as described in our other responses below.

**Reviewer #2 (Public review):**

The present study, led by Thomas and collaborators, aims to describe the firing activity of individual motor units in mice during locomotion. To achieve this, they implanted small arrays of eight electrodes in two heads of the triceps and performed spike sorting using a custom implementation of Kilosort. Simultaneously, they tracked the positions of the shoulder, elbow, and wrist using a single camera and a markerless motion capture algorithm (DeepLabCut). Repeated one-minute recordings were conducted in six mice at five different speeds, ranging from 10 to 27.5 cm·s⁻¹.

From these data, the authors reported that:

(1) a significant portion of the identified motor units was not consistently recruited across strides,

(2) motor units identified from the lateral head of the triceps tended to be recruited later than those from the long head,

(3) the number of spikes per stride and peak firing rates were correlated in both muscles, and

(4) the probability of motor unit recruitment and firing rates increased with walking speed.

The authors conclude that these differences can be attributed to the distinct functions of the muscles and the constraints of the task (i.e., speed).

Strengths:

The combination of novel electrode arrays to record intramuscular electromyographic signals from a larger muscle volume with an advanced spike sorting pipeline capable of identifying populations of motor units.

We thank the Reviewer for this comment.

Weaknesses:

(1) There is a lack of information on the number of identified motor units per muscle and per animal.

The Reviewer is correct that this information was not explicitly provided in the prior submission. We have therefore added Table 1 that quantifies the number of motor units per muscle and per animal.

(2) All identified motor units are pooled in the analyses, whereas per-animal analyses would have been valuable, as motor units within an individual likely receive common synaptic inputs. Such analyses would fully leverage the potential of identifying populations of motor units.

Please see our answer to the following point, where we address questions (2) and (3) together.

(3) The current data do not allow for determining which motor units were sampled from each pool. It remains unclear whether the sample is biased toward high-threshold motor units or representative of the full pool.

We thank the Reviewer for these comments. To clarify how motor unit responses were distributed across animals and muscle targets, we updated or added the following figures:

Figure 2C

Figure 4–figure supplement 1

Figure 5–figure supplement 2

Figure 6–figure supplement 2

These provide a more complete look at the range of activity within each motor pool, suggesting that we do measure from units with different activation thresholds within the same motor pool, rather than this variation being due to cross-animal differences. For example, Figure 2C illustrates that motor units from the same muscle and animal show a wide variety of recruitment probabilities. However, the limited number of motor units recorded from each individual animal does not allow a statistically rigorous test for examining cross-animal differences.

(4) The behavioural analysis of the animals relies solely on kinematics (2D estimates of elbow angle and stride timing). Without ground reaction forces or shoulder angle data, drawing functional conclusions from the results is challenging.

The Reviewer is correct that we did not measure muscular force generation or ground reaction forces in the present study. Although outside the scope of this study, future work might employ buckle force transducers as used in larger animals (Biewener et al., 1988; Karabulut et al., 2020) to examine the complex interplay between neural commands, passive biomechanics, and the complex force-generating properties of muscle tissue.

Major comments:

(1) Spike sorting

The conclusions of the study rely on the accuracy and robustness of the spike sorting algorithm during a highly dynamic task. Although the pipeline was presented in a previous publication (Chung et al., 2023, eLife), a proper validation of the algorithm for identifying motor unit spikes is still lacking. This is particularly important in the present study, as the experimental conditions involve significant dynamic changes. Under such conditions, muscle geometry is altered due to variations in both fibre pennation angles and lengths.

This issue differs from electrode drift, and it is unclear whether the original implementation of Kilosort includes functions to address it. Could the authors provide more details on the various steps of their pipeline, the strategies they employed to ensure consistent tracking of motor unit action potentials despite potential changes in action potential waveforms, and the methods used for manual inspection of the spike sorting algorithm's output?

This is an excellent point and we agree that the dynamic behavior used in this investigation creates potential new challenges for spike sorting. In our analysis, Kilosort 2.5 provides key advantages in comparing unit waveforms across multiple channels and in detecting overlapping spikes. We modified this version of Kilosort to construct unit waveform templates using only the channels within the same muscle (Chung et al., 2023), as clarified in the revised Methods section (see “Electromyography (EMG)”):

“A total of 33 units were identified across all animals. Each unit’s isolation was verified by confirming that no more than 2% of inter-spike intervals violated a 1 ms refractory limit. Additionally, we manually reviewed cross-correlograms to ensure that each waveform was only reported as a single motor unit.”

The Reviewer is correct that our ability to precisely measure a unit’s activity based on its waveform will depend on the relationship between the embedded electrode and the muscle geometry, which alters over the course of the stride. As a follow-up to the original text, we have included new analyses to characterize the waveform activity throughout the experiment and stride (also in Methods):

“We further validated spike sorting by quantifying the stability of each unit’s waveform across time (Figure 1–figure supplement 1). First, we calculated the median waveform of each unit across every trial to capture long-term stability of motor unit waveforms. Additionally, we calculated the median waveform through the stride binned in 50 ms increments using spiking from a single trial. This second metric captures the stability of our spike sorting during the rapid changes in joint angles that occur during the burst of an individual motor unit. In doing so, we calculated each motor unit’s waveforms from the single channel in which that unit’s amplitude was largest and did not attempt to remove overlapping spikes from other units before measuring the median waveform from the data. We then calculated the correlation between a unit’s waveform over either trials or bins in which at least 30 spikes were present. The high correlation of a unit waveform over time, despite potential changes in the electrodes’ position relative to muscle geometry over the dynamic task, provides additional confidence in both the stability of our EMG recordings and the accuracy of our spike sorting.”

We have included a supplementary to Figure 1 to highlight the effectiveness of our spike sorting.

(2) Yield of the spike sorting pipeline and analyses per animal/muscle

A total of 33 motor units were identified from two heads of the triceps in six mice (17 from the long head and 16 from the lateral head). However, precise information on the yield per muscle per animal is not provided. This information is crucial to support the novelty of the study, as the authors claim in the introduction that their electrode arrays enable the identification of populations of motor units. Beyond reporting the number of identified motor units, another way to demonstrate the effectiveness of the spike sorting algorithm would be to compare the recorded EMG signals with the residual signal obtained after subtracting the action potentials of the identified motor units, using a signal-to-residual ratio.

Furthermore, motor units identified from the same muscle and the same animal are likely not independent due to common synaptic inputs. This dependence should be accounted for in the statistical analyses when comparing changes in motor unit properties across speeds and between muscles.

We thank the Reviewer for this comment. Regarding motor unit yield, as described above the newly-added Table 1 displays the yield from each animal and muscle.

Regarding spike sorting, while signal-to-residual is often an excellent metric, it is not ideal for our high-resolution EMG signals since isolated single motor units are typically superimposed on a “bulk” background consisting of the low-amplitude waveforms of other motor units. Because these smaller units typically cannot be sorted, it is challenging to estimate the “true” residual after subtracting (only) the largest motor unit, since subtracting each sorted unit’s waveform typically has a very small effect on the RMS of the total EMG signal. To further address concerns regarding spike sorting quality, we added Figure 1–figure supplement 1 that demonstrates motor units’ consistency over the experiment, highlighting that the waveform maintains its shape within each stride despite muscle/limb dynamics and other possible sources of electrical noise or artifact.

Finally, the Reviewer is correct that individual motor units in the same muscle are very likely to receive common synaptic inputs. These common inputs may reflect in sparse motor units being recruited in overlapping rather than different strides. Indeed, in the following text added to the Results, we identified that motor units are recruited with higher probability when additional units are recruited.

“Probabilistic recruitment is correlated across motor units

Our results show that the recruitment of individual motor units is probabilistic even within a single speed quartile (Figure 5A-C) and predicts body movements (Figure 6), raising the question of whether the recruitment of individual motor units are correlated or independent. Correlated recruitment might reflect shared input onto the population of motor units innervating the muscle (De Luca, 1985; De Luca & Erim, 1994; Farina et al., 2014). For example, two motor units, each with low recruitment probabilities, may still fire during the same set of strides. To assess the independence of motor unit recruitment across the recorded population, we compared each unit’s empirical recruitment probability across all strides to its conditional recruitment probability during strides in which another motor unit from the same muscle was recruited (Figure 7). Doing this for all motor unit pairs revealed that motor units in both muscles were biased towards greater recruitment when additional units were active (p<0.001, Wilcoxon signed-rank tests for both the lateral and long heads of triceps). This finding suggests that probabilistic recruitment reflects common synaptic inputs that covary together across locomotor strides.”

(3) Representativeness of the sample of identified motor units

However, to draw such conclusions, the authors should exclusively compare motor units from the same pool and systematically track violations of the recruitment order. Alternatively, they could demonstrate that the motor units that are intermittently active across strides correspond to the smallest motor units, based on the assumption that these units should always be recruited due to their low activation thresholds.

One way to estimate the size of motor units identified within the same muscle would be to compare the amplitude of their action potentials, assuming that all motor units are relatively close to the electrodes (given the selectivity of the recordings) and that motoneurons innervating more muscle fibres generate larger motor unit action potentials.

We thank the Reviewer for this comment. Below, we provide more detailed analyses of the relationships between motor unit spike amplitude and the recruitment probability as well as latency (relative to stride onset) of activation.

We generated Author response image 1 to illustrate the relationship between the amplitude of motor units and their firing properties. As suspected, units with larger-amplitude waveforms fired with lower probability and produced their first spikes later in the stride. If we were comfortable assuming that larger spike amplitudes mean higher-force units, then this would be consistent with a key prediction of the size principle (i.e. that higher-force units are recruited later). However, we are hesitant to base any conclusions on this assumption or emphasize this point with a main-text figure, since EMG signal amplitude may also vary due to the physical properties of the electrode and distance from muscle fibers. Thus it is possible that a large motor unit may have a smaller waveform amplitude relative to the rest of the motor pool.

**Author response image 1.** Relation between motor unit amplitude and (A) recruitment probability and (B) mean first spike time within the stride. Colored lines indicate the outcome of linear regression analyses.

Currently, the data seem to support the idea that motor units that are alternately recruited across strides have recruitment thresholds close to the level of activation or force produced during slow walking. The fact that recruitment probability monotonically increases with speed suggests that the force required to propel the mouse forward exceeds the recruitment threshold of these "large" motor units. This pattern would primarily reflect spatial recruitment following the size principle rather than flexible motor unit control.

We thank the Reviewer for this comment. We agree with this interpretation, particularly in relation to the references suggested in later comments, and have added the following text to the Discussion to better reflect this argument:

“To investigate the neuromuscular control of locomotor speed, we quantified speed-dependent changes in both motor unit recruitment and firing rate. We found that the majority of units were recruited more often and with larger firing rates at faster speeds (Figure 5, Figure5–figure supplement 1). This result may reflect speed-dependent differences in the common input received by populations of motor neurons with varying spiking thresholds (Henneman et al., 1965). In the case of mouse locomotion, faster speeds might reflect a larger common input, increasing the recruitment probability as more neurons, particularly those that are larger and generate more force, exceed threshold for action potentials (Farina et al., 2014).”

(4) Analysis of recruitment and firing rates

The authors currently report active duration and peak firing rates based on spike trains convolved with a Gaussian kernel. Why not report the peak of the instantaneous firing rates estimated from the inverse of the inter-spike interval? This approach appears to be more aligned with previous studies conducted to describe motor unit behaviour during fast movements (e.g., Desmedt & Godaux, 1977, J Physiol; Van Cutsem et al., 1998, J Physiol; Del Vecchio et al., 2019, J Physiol).

We thank the Reviewer for this comment. In the revised Discussion (see ‘Firing rates in mouse locomotion compared to other species’) we reference several examples of previous studies that quantified spike patterns based on the instantaneous firing rate. We chose to report the peak of the smoothed firing rate because that quantification includes strides with zero spikes or only one spike, which occur regularly in our dataset (and for which ISI rate measures, which require two spikes to define an instantaneous firing rate, cannot be computed). Regardless, in the revised Figure 4B, we present an analysis that uses inter-spike intervals as suggested, which yielded similar ranges of firing rates as the primary analysis.

(5) Additional analyses of behaviour

The authors currently analyse motor unit recruitment in relation to elbow angle. It would be valuable to include a similar analysis using the angular velocity observed during each stride, re broadly, comparing stride-by-stride changes in firing rates with changes in elbow angular velocity would further strengthen the final analyses presented in the results section.

We thank the Reviewer for this comment. To address this, we have modified Figure 6 and the associated Supplemental Figures, to show relationships in unit activation with both the range of elbow extension and the range of elbow velocity for each stride. These new Supplemental Figures show that the trends shown in main text Figure 6C and 6E (which show data from all speed quartiles on the same axes) are also apparent in both the slower and faster quartiles individually, although single-quartile statistical tests (with smaller sample size than the main analysis) not reach statistical significance in all cases.

**Reviewer #3 (Public review):**

Summary:

Using the approach of Myomatrix recording, the authors report that:

(1) Motor units are recruited differently in the two types of muscles.

(2) Individual units are probabilistically recruited during the locomotion strides, whereas the population bulk EMG has a more reliable representation of the muscle.

(3) The recruitment of units was proportional to walking speed.

Strengths:

The new technique provides a unique data set, and the data analysis is convincing and well-performed.

We thank the Reviewer for the comment.

Weaknesses:

The implications of "probabilistical recruitment" should be explored, addressed, and analyzed further.

Comments:

One of the study's main findings (perhaps the main finding) is that the motor units are "probabilistically" recruited. The authors do not define what they mean by probabilistically recruited, nor do they present an alternative scenario to such recruitment or discuss why this would be interesting or surprising. However, on page 4, they do indicate that the recruitment of units from both muscles was only active in a subset of strides, i.e., they are not reliably active in every step.

If probabilistic means irregular spiking, this is not new. Variability in spiking has been seen numerous times, for instance in human biceps brachii motor units during isometric contractions (Pascoe, Enoka, Exp physiology 2014) and elsewhere. Perhaps the distinction the authors are seeking is between fluctuation-driven and mean-driven spiking of motor units as previously identified in spinal motor networks (see Petersen and Berg, eLife 2016, and Berg, Frontiers 2017). Here, it was shown that a prominent regime of irregular spiking is present during rhythmic motor activity, which also manifests as a positive skewness in the spike count distribution (i.e., log-normal).

We thank the Reviewer for this comment and have clarified several passages in response. The Reviewer is of course correct that irregular motor unit spiking has been described previously and may reflect motor neurons’ operating in a high-sensitivity (fluctuation-driven) regime. We now cite these papers in the Discussion (see ‘Firing rates in mouse locomotion compared to other species’). Additionally, the revision clarifies that “probabilistically” - as defined in our paper - refers only to the empirical observation that a motor unit spikes during only a subset of strides, either when all locomotor speeds are considered together (Figure 2) or separately (Figure 5A-C):

“Motor units in both muscles exhibited this pattern of probabilistic recruitment (defined as a unit’s firing on only a fraction of strides), but with differing distributions of firing properties across the long and lateral heads (Figure 2).”

“Our findings (Figure 4) highlight that even with the relatively high firing rates observed in mice, there are still significant changes in firing rate and recruitment probability across the spikes within bursts (Figure 4B) and across locomotor speeds (Figure 5F). Future studies should more carefully examine how these rapidly changing spiking patterns derive from both the statistics of synaptic inputs and intrinsic properties of motor neurons (Manuel & Heckman, 2011; Petersen & Berg, 2016; Berg, 2017).”

**Recommendations for the authors:**

**Reviewer #1 (Recommendations for the authors):**

As mentioned above, there are several issues with the statistics that need to be corrected to properly support the claims made in the paper.

The authors compare the fractions of MUs that show significant variation across locomotor speeds in their firing rate and recruitment probability. However, it is not statistically founded to compare the results of separate statistical tests based on different kinds of measurements and thus have unconstrained differences in statistical power. The comparison of the fractional changes in firing rates and recruitment across speeds that follow is helpful, though in truth, by contemporary standards, one would like to see error bars on these estimates. These could be generated using bootstrapping.

The Reviewer is correct, and we have revised the manuscript to better clarify which quantities should or should not be compared, including the following passage (see “Motor unit mechanisms of speed control” in Results):

“Speed-dependent increases in peak firing rate were therefore also present in our dataset, although in a smaller fraction of motor units (22/33) than changes in recruitment probability (31/33). Furthermore, the mean (± SE) magnitude of speed-dependent increases was smaller for spike rates (mean rate_fast_/rate_slow_ of 111% ± 20% across all motor units) than for recruitment probabilities (mean p(recruitment)_fast_/p(recruitment)_slow_ of 179% ± 3% across all motor units). While fractional changes in rate and recruitment probability are not readily comparable given their different upper limits, these findings could suggest that while both recruitment and peak rate change across speed quartiles, increased recruitment probability may play a larger role in driving changes in locomotor speed.”

The description in the Methods of the tests for variation in firing rates and recruitment probability across speeds are extremely hard to understand - after reading many times, it is still not clear what was done, or why the method used was chosen. In the main text, the authors quote p-values and then state "bootstrap confidence intervals," which is not a statistical test that yields a p-value. While there are mathematical relationships between confidence intervals and statistical tests such that a one-to-one correspondence between them can exist, the descriptions provided fall short of specifying how they are related in the present instance. For this reason, and those described in what follows, it is not clear what the p-values represent.

Next, the authors refer to fitting a model ("a Poisson distribution") to the data to estimate firing rate and recruitment probability, that the model results agree with their actual data, and that they then bootstrapped from the model estimates to get confidence intervals and compute p-values. Why do this? Why not just do something much simpler, like use the actual spike counts, and resample from those? I understand that it is hard to distinguish between no recruitment and just no spikes given some low Poisson firing rate, but how does that challenge the ability to test if the firing rates or the number of spiking MUs changes significantly across speeds? I can come up with some reasons why I think the authors might have decided to do this, but reasoning like this really should be made explicit.

In addition, the authors would provide an unambiguous description of the model, perhaps using an equation and a description of how it was fit. For the bootstrapping, a clear description of how the resampling was done should be included. The focus on peak firing rate instead of mean (or median) firing rate should also be justified. Since peaks are noisier, I would expect the statistical power to be lower compared to using the mean or median.

We thank the Reviewer for the comments and have revised and expanded our discussion of the statistical tests employed. We expanded and clarified our description of these techniques in the updated Methods section:

“Joint model of rate and recruitment

We modeled the recruitment probability and firing rate based on empirical data to best characterize firing statistics within the stride. Particularly, this allowed for multiple solutions to explain why a motor unit would not spike within a stride. From the empirical data alone, strides with zero spikes would have been assumed to have no recruitment of a unit. However, to create a model of motor unit activity that includes both recruitment and rate, it must be possible that a recruited unit can have a firing rate of zero. To quantify the firing statistics that best represent all spiking and non-spiking patterns, we modeled recruitment probability and peak firing rate along the following piecewise function:

Eq. 1: $P\left (Y=y\right)\, =\, \left (1-p\right)\, +\ p*e^{-\lambda },\, if\ y=0$

Eq. 2: $\ P\left (Y=y\right)\, =\, p*\frac{e^{-\lambda }\lambda ^{y}}{y!}\, ,\, if\ y> 0$

where *y* denotes the observed peak firing rate on a given stride (determined by convolving motor unit spike times with a Gaussian kernel as described above), *p* denotes the probability of recruitment, and *λ* denotes the expected peak firing rate from a Poisson distribution of outcomes. Thus, an inactive unit on a given stride may be the result of either non-recruitment or recruitment with a stochastically zero firing rate. The above equations were fit by minimizing the negative log-likelihood of the parameters given the data.”

“Permutation test for joint model of rate and recruitment and type 2 regression slopes

To quantify differences in firing patterns across walking speeds, we subdivided each mouse’s total set of strides into speed quartiles and calculated rate (𝜆, Eq. 1 and 2, Fig. 5A-C) and recruitment probability terms (*p*, Eq. 1 and 2, Fig. 5D-F) for each unit in each speed quartile. Here we calculated the difference in both the rate and recruitment terms across the fastest and slowest speed quartiles (*p*_fast_-*p*_slow_ and 𝜆_fast_-𝜆_slow_). To test whether these model parameters were significantly different depending on locomotor speed, we developed a null model combining strides from both the fastest and slowest speed quartiles. After pooling strides from both quartiles, we randomly distributed the pooled set of strides into two groups with sample sizes equal to the original slow and fast quartiles. We then calculated the null model parameters for each new group and found the difference between like terms. To estimate the distribution of possible differences, we bootstrapped this result using 1000 random redistributions of the pooled set of strides. Following the permutation test, the 95% confidence interval of this final distribution reflects the null hypothesis of no difference between groups. Thus, the null hypothesis can be rejected if the true difference in rate or recruitment terms exceeds this confidence interval.

We followed a similar procedure to quantify cross-muscle differences in the relationship between firing parameters. For each muscle, we estimated the slope across firing parameters for each motor unit using type 2 regression. In this case, the true difference was the difference in slopes between muscles. To test the null hypothesis that there was no difference in slopes, the null model reflected the pooled set of units from both muscles. Again, slopes were calculated for 1000 random resamplings of this pooled data to estimate the 95% confidence interval.”

The argument for delayed activation of the lateral head is interesting, but I am not comfortable saying the nervous system creates a delay just based on observations of the mean time of the first spike, given the potential for differential variability in spike timing across muscles and MUs. One way to make a strong case for a delay would be to show aggregate PSTHs for all the spikes from all the MUs for each of the two heads. That would distinguish between a true delay and more gradual or variable activation between the heads.

This is a good point and we agree that the claim made about the nervous system is too strong given the results. Even with Author response image 2 that the Reviewer suggested, there is still not enough evidence to isolate the role of the nervous system in the muscles’ activation.

**Author response image 2.** Aggregate peristimulus time histogram (PSTH) for all motor unit spike times in the long head (top) and lateral head (bottom) within the stride.

In the ideal case, we would have more simultaneous recordings from both muscles to make a more direct claim on the delay. Still, within the current scope of the paper, to correct this and better describe the difference in timing of muscle activity, we edited the text to the following:

“These findings demonstrate that despite the synergistic (extensor) function of the long and lateral heads of the triceps at the elbow, the motor pool for the long head becomes active roughly 100 ms before the motor pool supplying the lateral head during locomotion (Figure 3C).”

The results from Marshall et al. 2022 suggest that the recruitment of some MUs is not just related to muscle force, but also the frequency of force variation - some of their MUs appear to be recruited only at certain frequencies. Figure 5C could have shown signs of this, but it does not appear to. We do not really know the force or its frequency of variation in the measurements here. I wonder whether there is additional analysis that could address whether frequency-dependent recruitment is present. It may not be addressable with the current data set, but this could be a fruitful direction to explore in the future with MU recordings from mice.

We agree that this would be a fruitful direction to explore, however the Reviewer is correct that this is not easily addressable with the dataset. As the Reviewer points out, stride frequency increases with increased speed, potentially offering the opportunity to examine how motor unit activity varies with the frequency, phase, and amplitude of locomotor movements. However, given our lack of force data (either joint torques or ground reaction forces), dissociating the frequency/phase/amplitude of skeletal kinematics from the frequency/phase/amplitude of muscle force. Marshall et al. (2022) mitigated these issues by using an isometric force-production task (Marshall et al., 2022). Therefore, while we agree that it would be a major contribution to extend such investigations to whole-body movements like locomotion, given the complexities described above we believe this is a project for the future, and beyond the scope of the present study.

Minor:

Page 5: "Units often displayed no recruitment in a greater proportion of strides than for any particular spike count when recruited (Figures 2A, B)," - I had to read this several times to understand it. I suggest rephrasing for clarity.

We have changed the text to read:

“Units demonstrated a variety of firing patterns, with some units producing 0 spikes more frequently than any non-zero spike count (Figure 2A, B),...”

Figure 3 legend: "Mean phase ({plus minus} SE) of motor unit burst duration across all strides.": It is unclear what this means - durations are not usually described as having a phase. Do we mean the onset phase?

We have changed the text to read:

“Mean phase ± SE of motor unit burst activity within each stride”

Page 9: "suggesting that the recruitment of individual motor units in the lateral and long heads might have significant (and opposite) effects on elbow angle in strides of similar speed (see Discussion)." I wouldn't say "opposite" here - that makes it sound like the authors are calling the long head a flexor. The authors should rephrase or clarify the sense in which they are opposite.

This is a fair point and we agree we should not describe the muscles as ‘opposite’ when both muscles are extensors. We have removed the phrase ‘and opposite’ from the text.

Page 11: "in these two muscles across in other quadrupedal species" - typo.

We have corrected this error.

Page 16: This reviewer cannot decipher after repeated attempts what the first two sentences of the last paragraph mean. - “Future studies might also use perturbations of muscle activity to dissociate the causal properties of each motor unit’s activity from the complex correlation structure of locomotion. Despite the strong correlations observed between motor unit recruitment and limb kinematics (Fig. 6, Supplemental Fig. 3), these results might reflect covariations of both factors with locomotor speed rather than the causal properties of the recorded motor unit.”

For better clarity, we have changed the text to read:

“Although strong correlations were observed between motor unit recruitment and limb kinematics during locomotion (Figure 6, Figure 6–figure supplement 1), it remains unclear whether such correlations actually reflect the causal contributions that those units make to limb movement. To resolve this ambiguity, future studies could use electrical or optical perturbations of muscle contraction levels (Kim et al., 2024; Lu et al., 2024; Srivastava et al., 2015, 2017) to test directly how motor unit firing patterns shape locomotor movements.The short-latency effects of patterned motor unit stimulation (Srivastava et al., 2017) could then reveal the sensitivity of behavior to changes in muscle spiking and the extent to which the same behaviors can be performed with many different motor commands.”

**Reviewer #2 (Recommendations for the authors):**

Minor comments:

Introduction:

(1) "Although studies in primates, cats, and zebrafish have shown that both the number of active motor units and motor unit firing rates increase at faster locomotor speeds (Grimby, 1984; Hoffer et al., 1981, 1987; Marshall et al., 2022; Menelaou & McLean, 2012)." I would remove Marshall et al. (2022) as their monkeys performed pulling tasks with the upper limb. You can alternatively remove locomotor from the sentence and replace it with contraction speed.

Thank you for the comment. While we intended to reference this specific paper to highlight the rhythmic activity in muscles, we agree that this deviates from ‘locomotion’ as it is referenced in the other cited papers which study body movement. We have followed the Reviewer’s suggestion to remove the citation to Marshall et al.

(2) "The capability and need for faster force generation during dynamic behavior could implicate motor unit recruitment as a primary mechanism for modulating force output in mice."

The authors could add citations to this sentence, of works that showed that recruitment speed is the main determinant of the rate of force development (see for example Dideriksen et al. (2020) J Neurophysiol; J. L. Dideriksen, A. Del Vecchio, D. Farina, Neural and muscular determinants of maximal rate of force development. J Neurophysiol 123, 149-157 (2020)).

Thank you for pointing out this important reference. We have included this as a citation as recommended.

Results:

(3) "Electrode arrays (32-electrode Myomatrix array model RF-4x8-BHS-5) were implanted in the triceps brachii (note that Figure 1D shows the EMG signal from only one of the 16 bipolar recording channels), and the resulting data were used to identify the spike times of individual motor units (Figure 1E) as described previously (Chung et al., 2023)."

This sentence can be misleading for the reader as the array used by the researchers has 4 threads of 8 electrodes. Would it be possible to specify the number of electrodes implanted per head of interest? I assume 8 per head in most mice (or 4 bipolar channels), even if that's not specifically written in the manuscript.

Thank you for the suggestion. As described above, we have added Table 1, which includes all array locations, and we edited the statement referenced in the comment as follows:

“Electrode arrays (32-electrode Myomatrix array model RF-4x8-BHS-5) were implanted in forelimb muscles (note that Figure 1D shows the EMG signal from only one of the 16 bipolar recording channels), and the resulting data were used to identify the spike times of individual motor units in the triceps brachii long and lateral heads (Table 1, Figure 1E) as described previously (Chung et al., 2023).“

(4) "These findings demonstrate that despite the overlapping biomechanical functions of the long and lateral heads of the triceps, the nervous system creates a consistent, approximately 100 ms delay (Figure 3C) between the activation of the two muscles' motor neuron pools. This timing difference suggests distinct patterns of synaptic input onto motor neurons innervating the lateral and long heads."

Both muscles don't have fully overlapping biomechanical functions, as one of them also acts on the shoulder joint. Please be more specific in this sentence, saying that both muscles are synergistic at the elbow level rather than "have overlapping biomechanical functions".

We agree with the above reasoning and that our manuscript should be clearer on this point. We edited the above text in accordance with the Reviewer suggestion as follows:

"These findings demonstrate that despite the synergistic (extensor) function of the long and lateral heads of the triceps at the elbow, …”

(5) "Together with the differences in burst timing shown in Figure 3B, these results again suggest that the motor pools for the lateral and long heads of the triceps receive distinct patterns of synaptic input, although differences in the intrinsic physiological properties of motor neurons innervating the two muscles might also play an important role."

It is difficult to draw such an affirmative conclusion on the synaptic inputs from the data presented by the authors. The differences in firing rates may solely arise from other factors than distinct synaptic inputs, such as the different intrinsic properties of the motoneurons or the reception of distinct neuromodulatory inputs.

To better explain our findings, we adjusted the above text in the Results (see “Motor unit firing patterns in the long and lateral heads of the triceps”):

“Together with the differences in burst timing shown in Figure 3B, these results again suggest that the motor pools for the lateral and long heads of the triceps receive distinct patterns of synaptic input, although differences in the intrinsic physiological properties of motor neurons innervating the two muscles might also play an important role.”

We also included the following distinction in the Discussion (see “Differences in motor unit activity patterns across two elbow extensors”) to address the other plausible mechanisms mentioned.

“The large differences in burst timing and spike patterning across the muscle heads suggest that the motor pools for each muscle receive distinct inputs. However, differences in the intrinsic physiological properties of motor units and neuromodulatory inputs across motor pools might also make substantial contributions to the structure of motor unit spike patterns (Martínez-Silva et al., 2018; Miles & Sillar, 2011).”

(6) "We next examined whether the probabilistic recruitment of individual motor units in the triceps and elbow extensor muscle predicted stride-by-stride variations in elbow angle kinematics."

I'm not sure that the wording is appropriate here. The analysis does not predict elbow angle variations from parameters extracted from the spiking activity. It rather compares the average elbow angle between two conditions (motor unit active or not active).

We thank the Reviewer for this comment and agree that the wording could be improved here to better reflect our analysis. To lower the strength of our claim, we replaced usage of the word

‘predict’ with ‘correlates’ in the above text and throughout the paper when discussing this result.

Methods:

(7) "Using the four threads on the customizable Myomatrix array (RF-4x8-BHS-5), we implanted a combination of muscles in each mouse, sometimes using multiple threads within the same muscle. [...] Some mice also had threads simultaneously implanted in their ipsilateral or contralateral biceps brachii although no data from the biceps is presented in this study."

A precise description of the localisation of the array (muscles and the number of arrays per muscle) for each animal would be appreciated.

(8) "A total of 33 units were identified and manually verified across all animals." A precise description of the number of motor units concurrently identified per muscle and per animal would be appreciated. Moreover, please add details on the manual inspection. Does it involve the manual selection of missing spikes? What are the criteria for considering an identified motor unit as valid?

As discussed earlier, we added Table 1 to the main text to provide the details mentioned in the above comments.

Regarding spike sorting, given the very large number of spikes recorded, we did not rely on manual adjusting mislabeled spikes. Instead, as described in the revised Methods section, we verified unit isolation by ensuring units had >98% of spikes outside of 1ms of each other. Moreover, as described above we have added new analyses (Figure 1–figure supplement 1) confirming the stability of motor unit waveforms across both the duration of individual recording sessions (roughly 30 minutes) and across the rapid changes in limb position within individual stride cycles (roughly 250 msec).

**Reviewer #3 (Recommendations for the authors):**

Figure 2 (and supplement) show spike count distributions with strong positive skewness, which is in accordance with the prediction of a fluctuation-driven regime. I suggest plotting these on a logarithmic x-axis (in addition to the linear axis), which should reveal a bell-shaped distribution, maybe even Gaussian, in a majority of the units.

We thank the Reviewer for the suggestion. We present the requested analysis (Author response image 3), which shows bell-shaped distributions for some (but not all) distributions. However, we believe that investigating why some replotted distributions are Gaussian and others are not falls beyond the scope of this paper, and likely requires a larger dataset than the one we were able to obtain.

**Author response image 3.** Spike count distributions for each motor unit on a logarithmic x-axis.

Why not more data? I tried to get an overview of how much data was collected.

Supplemental Figure 1 has all the isolated units, which amounts to 38 (are the colors the two muscle types?). Given there are 16 leads in each myomatrix, in two muscles, of six mice, this seems like a low yield. Could the authors comment on the reasons for this low yield?

Regarding motor unit yield, even with multiple electrodes per muscle and a robust sorting algorithm, we often isolated only a few units per muscle. This yield likely reflects two factors. First, because of the highly dynamic nature of locomotion and high levels of muscle contraction, isolating individual spikes reliably across different locomotor speeds is inherently challenging, regardless of the algorithm being employed. Second, because the results of spike-train analyses can be highly sensitive to sorting errors, we have only included the motor units that we can sort with the highest possible confidence across thousands of strides.

Minor:

Figure captions especially Figure 6: The text is excessively long. Can the text be shortened?

We thank the Reviewer for this comment. Generally, we seek to include a description of the methods and results within the figure captions, but we concede that we can condense the information in some cases. In a number of cases, we have moved some of the descriptive text from the caption to the Methods section.

References

Berg, R. W. (2017). Neuronal Population Activity in Spinal Motor Circuits: Greater Than the Sum of Its Parts. Frontiers in Neural Circuits, 11. https://doi.org/10.3389/fncir.2017.00103

Biewener, A. A., Blickhan, R., Perry, A. K., Heglund, N. C., & Taylor, C. R. (1988). Muscle Forces During Locomotion in Kangaroo Rats: Force Platform and Tendon Buckle Measurements Compared. Journal of Experimental Biology, 137(1), 191–205. https://doi.org/10.1242/jeb.137.1.191

Chung, B., Zia, M., Thomas, K. A., Michaels, J. A., Jacob, A., Pack, A., Williams, M. J., Nagapudi, K., Teng, L. H., Arrambide, E., Ouellette, L., Oey, N., Gibbs, R., Anschutz, P., Lu, J., Wu, Y., Kashefi, M., Oya, T., Kersten, R., … Sober, S. J. (2023). Myomatrix arrays for high-definition muscle recording. eLife, 12, RP88551. https://doi.org/10.7554/eLife.88551

De Luca, C. J. (1985). Control properties of motor units. Journal of Experimental Biology, 115(1), 125–136. https://doi.org/10.1242/jeb.115.1.125

De Luca, C. J., & Erim, Z. (1994). Common drive of motor units in regulation of muscle force. Trends in Neurosciences, 17(7), 299–305. https://doi.org/10.1016/0166-2236(94)90064-7

Farina, D., Negro, F., & Dideriksen, J. L. (2014). The effective neural drive to muscles is the common synaptic input to motor neurons. The Journal of Physiology, 592(16), 3427–3441. https://doi.org/10.1113/jphysiol.2014.273581

Hartigan, P. M. (1985). Algorithm AS 217: Computation of the Dip Statistic to Test for Unimodality. Applied Statistics, 34(3), 320. https://doi.org/10.2307/2347485

Henneman, E., Somjen, G., & Carpenter, D. O. (1965). FUNCTIONAL SIGNIFICANCE OF CELL SIZE IN SPINAL MOTONEURONS. Journal of Neurophysiology, 28(3), 560–580. https://doi.org/10.1152/jn.1965.28.3.560

Karabulut, D., Dogru, S. C., Lin, Y.-C., Pandy, M. G., Herzog, W., & Arslan, Y. Z. (2020). Direct Validation of Model-Predicted Muscle Forces in the Cat Hindlimb During Locomotion. Journal of Biomechanical Engineering, 142(5), 051014. https://doi.org/10.1115/1.4045660

Kim, J. J., Wyche, I. S., Olson, W., Lu, J., Bakir, M. S., Sober, S. J., & O’Connor, D. H. (2024). Myo-optogenetics: Optogenetic stimulation and electrical recording in skeletal muscles. https://doi.org/10.1101/2024.06.21.600113

Lu, J., Zia, M., Baig, D. A., Yan, G., Kim, J. J., Nagapudi, K., Anschutz, P., Oh, S., O’Connor, D., Sober, S. J., & Bakir, M. S. (2024). Opto-Myomatrix: μLED integrated microelectrode arrays for optogenetic activation and electrical recording in muscle tissue. https://doi.org/10.1101/2024.07.01.601601

Manuel, M., & Heckman, C. J. (2011). Adult mouse motor units develop almost all of their force in the subprimary range: A new all-or-none strategy for force recruitment? Journal of Neuroscience, 31(42), 15188–15194. https://doi.org/10.1523/JNEUROSCI.2893-11.2011

Marshall, N. J., Glaser, J. I., Trautmann, E. M., Amematsro, E. A., Perkins, S. M., Shadlen, M. N., Abbott, L. F., Cunningham, J. P., & Churchland, M. M. (2022). Flexible neural control of motor units. Nature Neuroscience, 25(11), 1492–1504. https://doi.org/10.1038/s41593-022-01165-8

Martínez-Silva, M. de L., Imhoff-Manuel, R. D., Sharma, A., Heckman, C. J., Shneider, N. A., Roselli, F., Zytnicki, D., & Manuel, M. (2018). Hypoexcitability precedes denervation in the large fast-contracting motor units in two unrelated mouse models of ALS. eLife, 7(2007), 1–26. https://doi.org/10.7554/eLife.30955

Miles, G. B., & Sillar, K. T. (2011). Neuromodulation of Vertebrate Locomotor Control Networks. Physiology, 26(6), 393–411. https://doi.org/10.1152/physiol.00013.2011

Petersen, P. C., & Berg, R. W. (2016). Lognormal firing rate distribution reveals prominent fluctuation–driven regime in spinal motor networks. eLife, 5. https://doi.org/10.7554/elife.18805

Srivastava, K. H., Elemans, C. P. H., & Sober, S. J. (2015). Multifunctional and Context-Dependent Control of Vocal Acoustics by Individual Muscles. The Journal of Neuroscience, 35(42), 14183–14194. https://doi.org/10.1523/JNEUROSCI.3610-14.2015

Srivastava, K. H., Holmes, C. M., Vellema, M., Pack, A. R., Elemans, C. P. H., Nemenman, I., & Sober, S. J. (2017). Motor control by precisely timed spike patterns. Proceedings of the National Academy of Sciences of the United States of America, 114(5), 1171–1176. https://doi.org/10.1073/pnas.1611734114
